# Increase in Hepatitis A Cases Linked to Imported Strains to Rio de Janeiro, Brazil: A Cross-Sectional Study

**DOI:** 10.3390/v14020303

**Published:** 2022-02-01

**Authors:** Vinicius M. Mello, Lucas M. Bianchi, Paulo Sergio F. Sousa, Pedro S. Tavares, Daniel R. G. Di Salvo, Cleber F. Ginuino, Nathalia A. A. Almeida, Carlos A. S. Fernandes, Francisco C. A. Mello, Livia M. Villar, Lia L. Lewis-Ximenez, Barbara V. Lago

**Affiliations:** 1 Viral Hepatitis Laboratory, Oswaldo Cruz Institute, Oswaldo Cruz Foundation, Rio de Janeiro 21040-900, Brazil; vmello.fiocruz@gmail.com (V.M.M.); paulosfsousa@gmail.com (P.S.F.S.); cleber@ioc.fiocruz.br (C.F.G.); fcamello@gmail.com (F.C.A.M.); liviafiocruz@gmail.com (L.M.V.); lialewis.fiocruz@gmail.com (L.L.L.-X.); 2 Sergio Arouca National School of Public Health, Oswaldo Cruz Foundation, Rio de Janeiro 21041-210, Brazil; lucas_bianchi123@hotmail.com; 3 Research Group on Territory and Politics, Geosciences Institute, Federal University of Rio de Janeiro, Rio de Janeiro 21941-916, Brazil; pedro_tava_sil@hotmail.com; 4 Cartography Laboratory, Geosciences Institute, Federal University of Rio de Janeiro, Rio de Janeiro 21941-916, Brazil; danieldisalvo97@gmail.com; 5 Molecular Virology Laboratory, Oswaldo Cruz Institute, Oswaldo Cruz Foundation, Rio de Janeiro 21040-900, Brazil; natybio92@yahoo.com.br; 6 Hepatitis Division, Rio de Janeiro Health State Secretariat, Rio de Janeiro 20211-110, Brazil; cas.fernandes@gmail.com; 7 Institute of Technology in Immunobiologics–Bio-Manguinhos, Oswaldo Cruz Foundation, Rio de Janeiro 21040-900, Brazil

**Keywords:** hepatitis A virus, hepatitis A in Brazil, molecular epidemiology, outbreak strains, endemics strains, sexual transmission

## Abstract

This study aims to evaluate the epidemiological and molecular features associated with HAV transmission in adults in Rio de Janeiro during a period of increased registered cases of HAV (2017–2018). Socio-epidemiological data and serum samples from anti-HAV IgM+ individuals were obtained. HAV RNA was RT-PCR amplified and sequenced for further phylogenetic and phylogeographic analyses. From fifty-two HAV IgM+ individuals, most were men (78.85%; *p* = 0.024), aged 20–30 years old (84.61%; *p* < 0.001), resided in the Rio de Janeiro north zone (31/52; 59.62%; *p* = 0.001), and are men who have sex with men (MSM) (57.69%; *p* = 0.002). Sexual practices were more frequent (96%) than others risk factors (food-borne (44%), water-borne (42.31%), and parenteral (34.62%)). Individuals who traveled to endemic regions had a 7.19-fold (1.93–36.04; *p* < 0.01) increased risk of HAV. Phylogenetic analysis revealed four distinct clades of subgenotype IA, three of them comprised sequences from European/Asian MSM outbreaks and one from Brazilian endemic strains. Bayesian Inference showed that the imported strains were introduced to Brazil during large mass sportive events. Sexual orientation and sexual practices may play a role in acquiring HAV infection. Public policies targeting key populations must be implemented to prevent further dissemination of HAV and other STIs.

## 1. Introduction

Hepatitis A virus (HAV) can cause a self-limited and acute disease [1]. The virus is transmitted through the fecal–oral route, mainly by the consumption of contaminated food and water [1,2]. However, its transmission can also occur through person-to-person contact. This contact may be subdivided into household contact (contact with infected patients and children) [1,2,3] and sexual contact (anal, oral, and oroanal sex) [1,2,3,4,5,6]. On rare occasions, the infection can also be transmitted through blood transfusion, blood products [7,8], or among injecting drug users [1,5,7].

Children in Brazil were predominantly affected by HAV infection before the inclusion of hepatitis A vaccination for infants in 2014, which resulted in a sharp decline in registered infected children following this new protocol [5,6,9,10]. However, besides the drop in registered cases among children, a shift in this infection profile has been observed, where young adults have been more affected [2,10]. This group represents 70–90% of symptomatic cases worldwide [5,9].

Several outbreaks in adults, mainly in men who have sex with men (MSM), related to sexual practices, have occurred around the world. Distinct strains of subgenotype IA (VRD_521_2016, RIVM-HAV16–090 and V16-25801) have been identified in the European Union, Asia, North and South Americas since 2016 [11,12,13,14,15,16]. In Brazil, an increase in the number of cases in the southeastern and southern regions from 2017 to 2019 has been observed following a decade of declining notifications [10,17]. In this setting, the Rio de Janeiro state showed a boost in HAV incidence from 0.3 cases per 100,000 inhabitants in 2016 to 9.0 cases in 2018, with the state capital holding most of these notifications [10,17,18]. Data from the Brazilian Ministry of Health showed an increase of 128% in the incidence of HAV infection in men aged 20–39 years in 2017 [17], and sexual practices were identified as the main route of transmission in this group [10,18].

In order to understand the factors associated with the transmission of HAV, this observational, analytical, and cross-sectional study aimed to assess risk behavior and molecular aspects in patients receiving medical care at the Viral Hepatitis Ambulatory, a reference clinic for acute viral hepatitis in Rio de Janeiro, during a period of increased registered cases of HAV (2017–2019). The results presented here identified the circulation of three HAV strains related to European/Asian outbreaks possibly introduced during sporting events in Rio de Janeiro, Brazil. Molecular characteristics and dissemination routes of HAV strains were discussed and dated.

## 2. Materials and Methods

### 2.1. Ethics

The Oswaldo Cruz Institute/IOC/FIOCRUZ Research Ethics Committee approved the study (number CAAE: 71396617.6.0000.5248 for samples from 2013–2016 and CAAE: 50230015.0.0000.5248 for samples from 2017–2019). All procedures were performed in accordance with the ethical standards of the responsible committee on human experimentation (institutional and national) and with the Helsinki Declaration of 1975, as revised in 2008. Samples from 2013–2016 were from the Laboratory of Viral Hepatitis (LAHEP) biorepository and were exempt from the consent form. All patients from 2017–2019 agreed to their participation in the research by signing the informed consent form.

### 2.2. Study Population

Admission criteria: All participants were adults (≥ 18 years old) followed up at the Viral Hepatitis Ambulatory during 2013-2019. Serum samples from individuals with HAV acute infection (defined as anti-HAV IgM positivity) from two periods were included in the study: (1) before the increase in HAV cases (2013–2016), and (2) during the HAV outbreak (2017–2019), from the Ambulatory and Central Public Health Laboratory of Rio de Janeiro. 

Samples from the period before the increase in cases (2013–2016) were used exclusively for molecular investigation, while samples from the outbreak period (2017–2019) were used for both molecular and epidemiological purposes. Furthermore, to understand the aspects related to the increased incidence of HAV infection in Rio de Janeiro between 2017 and 2019, an additional “control” group from the Viral Hepatitis Ambulatory was included for statistical analysis. The “control” group was composed of 87 non-HAV individuals (anti-HAV IgM negative) with other viral hepatitis. Active viral hepatitis was defined as: (1) elevated transaminases (above >33 IU/L for men and >25 IU/L for women); (2) with or without classical hepatitis clinical manifestations; (3) presence of serological and/or molecular markers for hepatitis B (HBV: n = 54; HBsAg+, anti-HBc+ and HBV DNA+), hepatitis C (HCV: n = 32; anti-HCV+ alone and/or anti-HCV+ plus HCV RNA+) or hepatitis E (HEV: n = 1; anti-HEV IgG+ and IgM+).

### 2.3. Socio-Epidemiological Data

The data collected from medical records included: gender, age, sexual orientation, and possible exposure factors for acquiring HAV infection. The exposure factors were divided into five major groups: (1) parenteral exposure; (2) consumption of contaminated food and/or water; (3) water-borne exposure; (4) sexual practices; and (5) travel to endemic regions. These factors were subdivided into several practices within each subcategory. Furthermore, information regarding residence location was collected, such as neighborhood and locality within Rio de Janeiro’s County Planning Area (divided inꓽ North, South, West, Central). For those not living in the city of Rio de Janeiro, data were obtained for the county of residence.

### 2.4. Specimens

Serum samples were obtained through the laboratory’s biorepository for phylogenetic and phylogeographic analyses of HAV strains. Anti-HAV IgM positive samples collected between 2013 and 2016 were randomly selected using the Excel^®®^ program version 1802 Build 9029.2167 (Microsoft Office©, Las Vegas, NV, USA), according to the following criteria: volume ≥ 1 mL and with a representative annual distribution, to contemplate both semesters of each year. For a better understanding and discussion of the phylogenetic analysis, we divided the sequenced samples into two groups called ‘endemic strains’ and ‘outbreak strains’. The ‘endemic strains’ were sequences identified before the period of increase in HAV infection (2013–2016), while the ‘outbreak strains’ were identified during the period of increase in HAV infection (2017–2019), linked to international strains described in outbreaks throughout the world.

All patients had been tested for hepatitis A using the commercial kit HAV IgM (LIAISON^®^ XL, DiaSorin, Saluggia, Italy). Additionally, these same patients had been tested for hepatitis B virus (HBV), hepatitis C virus (HCV), hepatitis E (HEV), and other sexually transmitted infections (STIs), such as human immunodeficiency virus (HIV) and syphilis, during their clinical evaluation at the clinic. Testing for the different STIs ranged from serological to rapid tests depending on availability. The following commercial assays were used: (1) HBV (tested for markersꓽ HBsAg and HBeAg antigen; anti-HBc IgM, anti-HBc IgG, anti-HBe and anti-HBs antibodies, using LIAISON^®^ XL, DiaSorin, Saluggia, Italy); (2) HCV (tested for Anti-HCV antibody, using LIAISON^®^ XL HCV Ab); (3) HEV (recomWell HEV IgG/IgM, Mikrogen, Neuried, Germany); (4) HIV (tested for Anti-HIV antibody and HIV antigens, using LIAISON^®^ MUREX HIV Ab/Ag, DiaSorin, Saluggia, Italy; and Immunoblot Rápido DPP^®^ HIV 1/2, Bio Manguinhos, Rio de Janeiro, Brazil), and (5) Syphilis (tested for Anti-Treponema, using Immuno-Rápido Sífilis^®^, Wama, São Paulo, Brazil).

### 2.5. Statistical Analysis

Descriptive statistics analyses of qualitative variables were determined by absolute frequency distribution, determined by the presence or absence of a risk factor for HAV infection. Subsequently, the Chi-square test was used for categorical variables, between the exposure and outcome variables, at 95% confidence intervals (CI) and *p* ≤ 0.05 to compare proportions between the data collected from the case and control groups.

A logistic regression model was performed to estimate the crude odds ratio estimates (OR), adjusted odds ratios (aOR), and their respective 95% CI to quantify the association between exposure variables and the outcome (defined as an individual positive for anti-HAV IgM), expressing the incidence for HAV infection. Statistical significance was set at a *p*-value ≤ 0.05. The variables included in the model were obtained from the five major groups of risk factors (parenteral exposure, ingestion of contaminated water and food, hydric exposure, sexual practices, and travel to endemic regions). The model selection was carried out based on the following adjustment criteria: first, the complete model, which included all categories of exposure to HAV. Subsequently, the importance of each category was tested, removing one category at a time to adjust the statistical model until the lowest value of the Akaike information criterion (AIC) was reached. At the end of the procedure, the best-reduced model was obtained. All analyses were performed using R statistical software version 4.0.3. (https://www.r-project.org/, accessed on 17 December 2021) [19].

### 2.6. HAV RNA Molecular Detection

HAV RNA was qualitatively detected by a reverse transcriptase PCR (RT-PCR) using the commercial kit SuperScript III reverse RT-PCR (Invitrogen, USA). Oligonucleotides used in both steps were described by De Paula and collaborators [20], amplifying a region with approximately 345 base pairs (bp) from the VP1-2A region of the HAV genome. The reaction was carried out using 5 μ RNA, 4.5 μL H_2_O DNAse/RNAse free, 12.5 μL 2× Reaction mix buffer, 1 μL RNAseOUT™ recombinant ribonuclease inhibitor (40 U/μL), 1 μL polymerase and 0.5 μL oligonucleotides sense and antisense. RT-PCR was composed of an initial cycle at 50 °C for 30 s, followed by a hybridization step at 94 °C for 2 min, and amplification for 5 cycles at 94 °C for 30 s, 50 °C for 30 s, and 68 °C for 1 min. Posteriorly, cDNA was amplified for 35 cycles at 94 °C for 30 s, 50 °C for 30 s with a drop of −0.3 °C per cycle, and 68 °C for 1 min, followed by an additional 5 min of extension at 68 °C in the last cycle.

Additionally, to increase the specificity and sensitivity of the reaction, a semi-nested PCR was performed. For this purpose, 2 μL of the RT-PCR product was used as a template and was added to a mix containing 37.8 μL of H_2_O DNAse/RNAse free, 1 μL dNTPs at 10 mM, 5 μL 10× Reaction Mix Buffer, 2 μL MgCl2 at 50 mM, 0.2 μL Platinum^®^ Taq DNA Polymerase (Invitrogen, Waltham, MA, USA), and 1 μL oligonucleotides sense and antisense, respectively. For this step, the cycle conditions were 94 °C for 2 min, 35 cycles at 94 °C for 30 s, 56 °C for 30 s, and 72 °C for 1 min, followed by an additional 5 min extension at 72 °C in the last cycle.

### 2.7. Nucleotide Sequencing, Phylogenetic, and Phylogeographic Analyses

Phylogenetic and Bayesian evolutionary analyses were conducted with a dataset composed of 65 VP1-2A reference sequences representing the main genotype IA involved in outbreaks worldwide, and Brazilian endemic strains with known collection dates retrieved from the GenBank (Table S1).

In order to mitigate possible location and temporal inference errors in spatiotemporal analyses, sequences retrieved from GenBank should follow the selection criteriaꓽ (1) have “sample collection place” and “year” filled correctly and (2) have been published in a scientific paper, where all the information of collection place and year can be confirmed.

The sequences obtained were analyzed with MEGA software version 10 (The Pennsylvania State University, USA) to determine the subgenotypes and access genetic diversity. Phylogenetic analysis was performed using the Maximum Likelihood method, under the General Time Reversible (GTR + G+I) substitution model (defined with the Model Selection tool as the best-fit model), with a 3000-replicate bootstrap resampling [21].

Calculations of the time of the most recent common ancestor (tMRCA) of internal nodes were estimated under an uncorrelated lognormal relaxed molecular clock. The Markov Chain Monte Carlo (MCMC) was run for 100 × 10^6^ generations using the General Time Reversible model with gamma-distributed rate heterogeneity (GTR + G+I) through the BEAST software version 1.8.10 (http://beast.community/, accessed on 17 December 2021), and their convergence (estimated sum of squares >200) were assessed using Tracer version 1.7. (http://beast.community/tracer, accessed on 17 December 2021). The uncertainties in the parameters were assessed by 95% highest posterior density (HPD) interval. The consensus tree was estimated by the TreeAnnotator program version 1.6.1 (https://beast.community/treeannotator, accessed on 17 December 2021) and visualized in the FigTree program version 1.4.3. (http://tree.bio.ed.ac.uk/software/figtree/, accessed on 17 December 2021) [22].

### 2.8. Map Construction

For the map construction, the city of Rio de Janeiro and the marked points were visualized in a Geographic Information System in ArcMap software version 10.1 (http://desktop.arcgis.com/en/arcmap/, accessed on 17 December 2021). The municipalities’ boundaries were extracted from the state cartographic base of the Foundation State Center for Statistics, Research and Training of Public Servants in Rio de Janeiro (CEPERJ Foundation) [23], and neighborhood boundaries were collected from the Pereira Passos Municipal Urbanism Institute (IPP) [24]. Geocentric Reference System for the Americas (SIRGAS 2000) [25] datum and cylindrical cartographic projection of the geodesic reference system were used.

## 3. Results

### 3.1. Samples Description and Socio-Demographic Characteristics and Case Distribution

**Figure 1:**
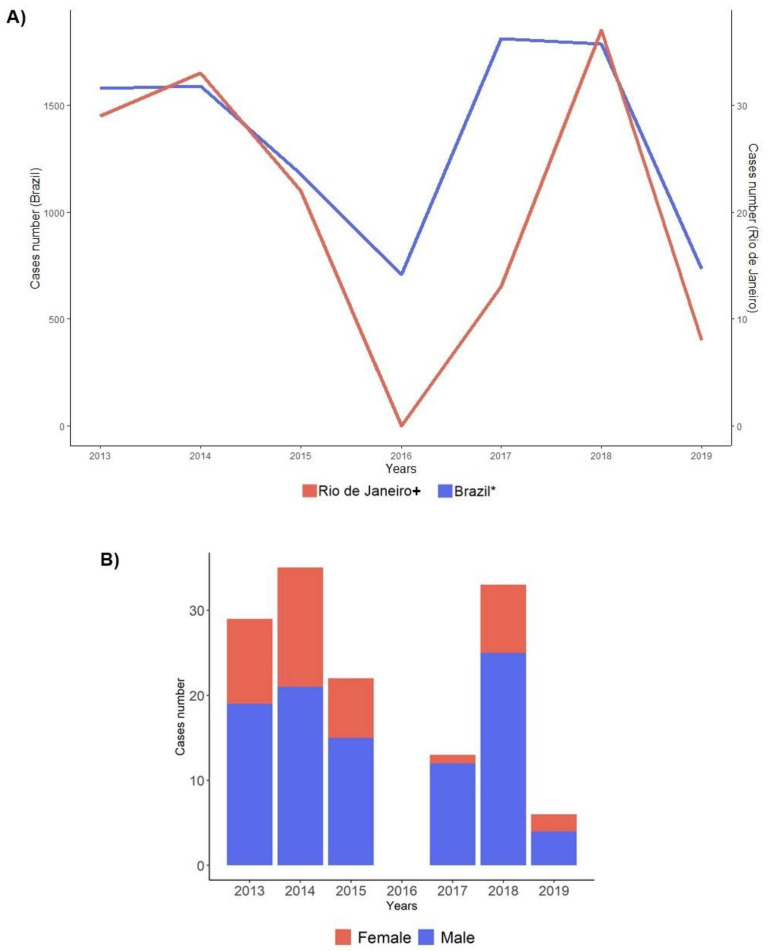
Distribution of notified HAV cases by year and gender per year. (**A**) Distribution of HAV registered cases in adults between 2013 and 2019 in Brazil and at the Viral Hepatitis Clinic/Rio de Janeiro. The blue line represents HAV cases registered in Brazil, and the red line represents cases registered in the Clinic. * Data kindly supplied by the General Coordination of Strategic Information, Department of Chronic Conditions and Sexually Transmitted Infections, Secretariat of Health Surveillance, Brazilian Ministry of Health. + Viral Hepatitis Ambulatory/ Viral Hepatitis Laboratory of the Oswaldo Cruz Institute, Oswaldo Cruz Foundation, Ministry of Health, Rio de Janeiro. (**B**) Gender distribution according to the year for cases followed at the Viral Hepatitis Ambulatory/Viral Hepatitis Laboratory of the Oswaldo Cruz Institute, Oswaldo Cruz Foundation, Ministry of Health, Rio de Janeiro. Blue color for the male gender and red for the female gender.

Altogether, 142 biological specimens from adult patients with acute HAV infection (anti-HAV IgM+) were investigated between 2013 and 2019, 84 before 2016 and 58 after 2016 (52 cases from the ambulatory and 6 from the state laboratory, corresponding to all adult cases in the outbreak period). Ages varied from 18 to 73 (average 30 years old), with the male gender representing 70.42% (100/142). The distribution of cases per year and gender is displayed in Figure 1.

Eighty-seven patients with non-HAV acute hepatitis infection (anti-HAV IgM negative) had complete socio-epidemiological data and were included as “controls” for the study. Data from these “controls” were obtained exclusively from patients followed up at the Ambulatory between 2017 and 2019 and compared with 52/58 HAV-infected “cases” collected in the same period who had successfully completed the socio-epidemiological questionnaire.

**Figure 2:**
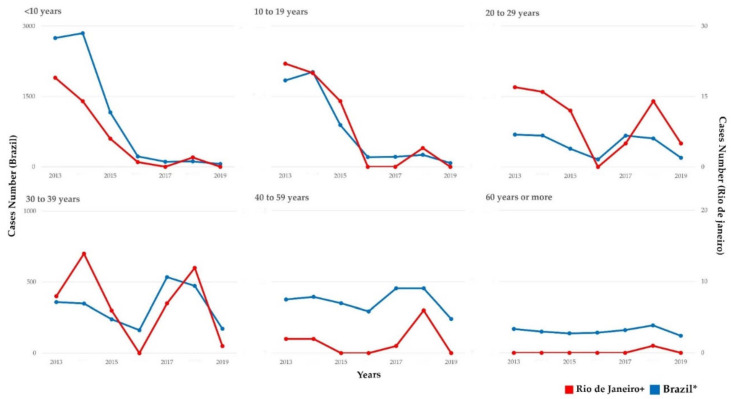
Distribution of HAV cases by years and age groups (2013–2019) in Brazil and the Viral Hepatitis Clinic/Rio de Janeiro. The red line represents the HAV cases registered in Brazil, and the blue line represents the cases registered in the Clinic. * Data kindly provided by the General Coordination of Strategic Information, Department of Chronic Conditions and Sexually Transmitted Infections, Secretariat of Health Surveillance, Ministry of Health of Brazil (can be found in the Boletim Epidemiologico Brasileiro [27]. + Viral Hepatitis Laboratory/Ambulatory, Instituto Oswaldo Cruz, Oswaldo Cruz Foundation, Ministry of Health, Rio de Janeiro.

**Figure 3:**
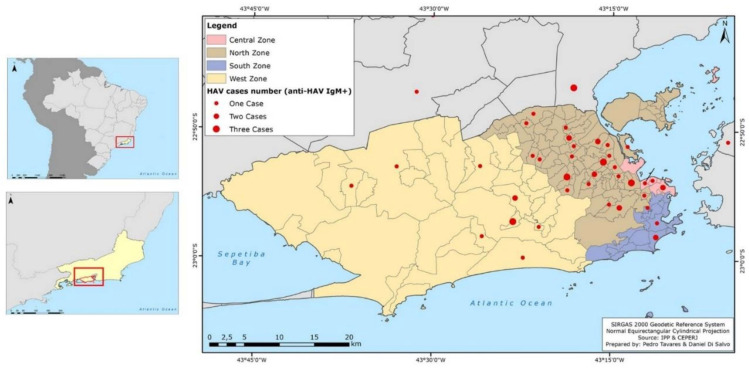
Distribution map of HAV cases (anti-IgM reagent) according to city/neighborhood/geographical zone of residence of patients attended at the clinic. Geographical zones are colored; North zone (brown); Central zone (light pink); South zone (blue); West zone (yellow), and neighboring cities (grey). The red dots (●) represent cases in each zone/neighborhood: small red dots represent one case, medium red dots two cases, and big red dots three cases. All information can be seen in the legend on the top left.

As shown in Figure 2, the distribution of HAV cases/year in Rio de Janeiro and Brazil displayed a similar profile, with most HAV cases concentrated in adult ages during the outbreak period. Statistical analysis showed that HAV-infected patients were mainly concentrated in the age group 20 to 29 years (24/52; 46.15%; *p* < 0.001). Male patients (41/52; 78.85%; *p* = 0.024) predominated, with a sex ratio of male and female of 12:1, 25:8, and 4:2 in the sampling from 2017, 2018, and 2019, respectively. As for sexual orientation, most individuals from the case group were MSM (30/52; 57.69%; *p* = 0.002). Individuals were primarily from the city of Rio de Janeiro (90.38%; 47/52), and from the north zone (31/52; 59.62%; 1.17 cases per 100,000 inhabitants; *p* = 0.001) (Table 1). Case distribution according to the county’s administrative regions and neighboring municipalities is demonstrated in Figure 3.

### 3.2. HAV Exposure Factors and Co-Infections

Sexual risk behavior was identified as the major risk factor and was reported in 96% (48/50) of the HAV cases. Most cases reported more than one type of risky sexual practice, such as oral sex (39/44; 88.64%), anal sex (34/46; 73.91%), and unprotected sex (36/46; 78.26%). According to aOR analysis, individuals who had traveled to endemic regions were 7.19 (1.93–36.04; *p* < 0.01) times more likely to become infected with HAV. When analyzing non-sexual risk factors, 34.62% (18/52) reported parenteral factors, 44% (22/50) food-borne factors, and 42.31% (22/52) water-borne factors, none of these categories having statistical significance (Table 2).

Regarding other STIs, 13/52 (25%) individuals were coinfected with HIV and/or syphilis (ten patients were infected with syphilis, seven of these additionally infected with HIV, and three infected exclusively with HIV). All were men (13/41; 31.7%) and MSM (13/30; 43.33%). In addition, 2/52 (3.8%) individuals were HCV coinfected, and 4/52 (7.69%) presented serological profiles compatible with HBV past infection. No individual presented HEV infection.

### 3.3. Viral Genome Detection, Genotyping, and Phylogenetic Analyses

A total of 77 HAV patients were enrolled in the molecular analysis, 19 from 2013–2016 and 58 from 2017–2019. HAV-RNA was detected in 62/77 (80.52%) IgM+ samples. For the 2013–2016 period, 17/19 samples tested HAV-RNA positive, five from 2013, five from 2014, and seven from 2015. No adult HAV cases were identified in 2016. As for the 2017–2019 period, HAV-RNA was detected in 45/58 samples, 10 from 2017, 35 from 2018, and 8 from 2019. Of the 62 detectable HAV-RNA samples, 52 (83.87%) were successfully sequenced.

**Figure 4:**
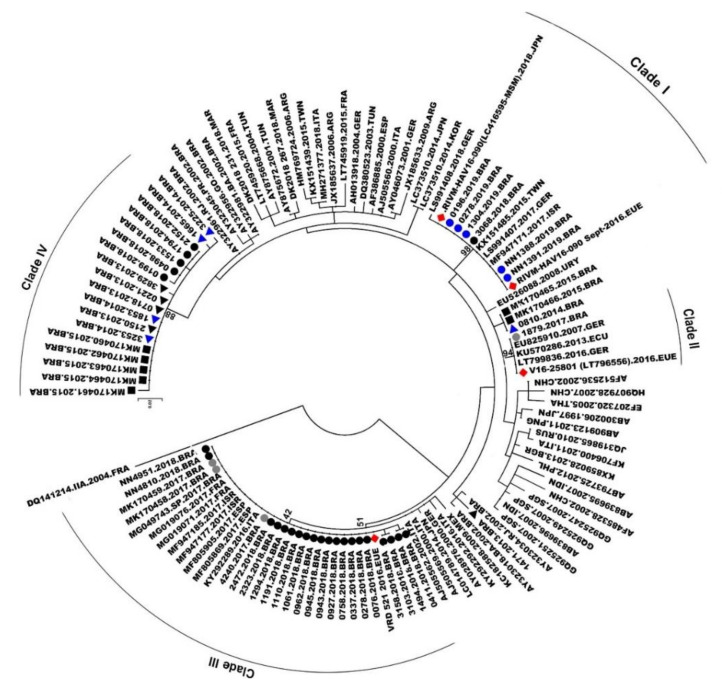
Phylogenetic tree generated using the Maximum Likelihood method with 3000-replicate bootstrap resampling, with 65 VP1-2A region reference sequences from genotype IA isolates from the GenBank. The number in the tree represents bootstraps. The viral European and Asian outbreak strains are marked with red rhombus (♦). The study sequences are marked with: 2013, black triangle (▲); 2014, blue triangle (▲); 2018, black dot (●); and 2019, blue dot (●). All study sequences are available on the GenBank; access numbers are available in Table S2. Sequences from 2015, black square (■), all previously published [15]; 2017, grey dot (●), two sequences published previously [15].

Phylogenetic analysis for the VP1/2A fragment showed that all 52 sequences belonged to genotype IA, 35 sequences from 2017–2019, and 17 from 2013–2015 (GenBank access numbers are available in Table S2). Sequenced samples clustered into four distinct monophyletic clades (named ‘Clade I to IV’) with high genetic identity and strong bootstrap support. These clades were classified into two distinct groups: outbreak strains (2017–2019), where sequences grouped with strains from recent European and Asian outbreaks (clades I, II, and III) (30/52; 57.7%); and endemic strains (2013–2015) (18/52; 34.61%) (sequences that grouped with Brazilian endemically circulating strains; clade IV). The majority (30/35; 85.7%) of the 2017–2019 sequences clustered in some epidemic clades. A few sequences from 2013–2015 (one from 2013 and one from 2014; and two from 2015) (4/52; 7.69%) did not cluster in any of these four main clades and were distributed in small, unrepresentative clades along the tree (Figure 4).

**Figure 5:**
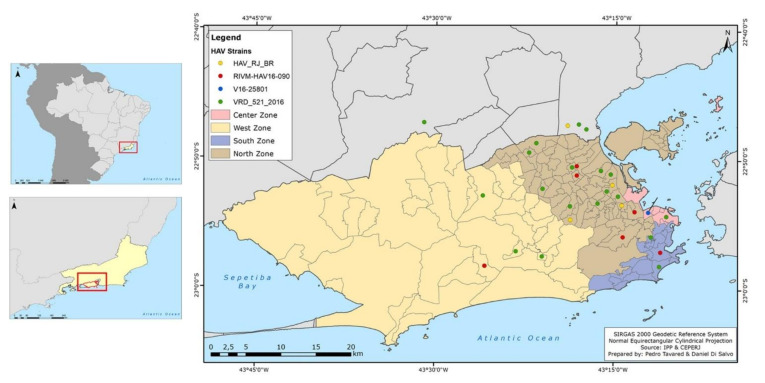
Distribution map of viral strains found in the study according to neighborhood/geographical area of Rio de Janeiro city. Geographical zones are colored: The viral strains found identified by colored dots: green (●) (VRD_521_2016 strain); red (●) (RIVM-HAV16-090 strains); blue (●) (V16-25801 strain); and yellow (●) Brazilian endemic strain (named as ‘HAV_RJ_BR’). All information can be seen in the legend on the top left.

A few sequences from 2018 (*n* = 1) and 2019 (*n* = 5) (6/52; 11.5%) were grouped in clade I with HAV European and Asian outbreaks strains (RIVM-HAV16-090_Sept and LC416569), presenting 100% of genetic identity. One sequence from 2017 (1/52; 1.9%) clustered with the European strain V16-25801 (clade II), with 99.99% genetic identity. Twenty-tree (*n* = 3 from 2017 and *n* = 20 from 2018) (23/52; 44.2%) sequences clustered in clade III, presenting 99.99% of identity with the European VRD_521_2016. Sequences from 2013 (*n* = 4), 2014 (*n* = 4), and 2015 (*n* = 5) clustered in clade IV (endemic clade) along with five sequences from 2018, showing 99.7% genetic identity. The viral strain distribution identified in the city of Rio de Janeiro is shown in Figure 5.

### 3.4. Phylogeographic Analyses and Bayesian Inference

The mean nucleotide substitution rate was estimated in 1.05 × 10^−5^ substitutions/site/year (95% HPD, 4.88 × 10^−4^ to 1.55 × 10^−3^). According to the Bayesian analysis (posterior probability (pp): 0.99), the most plausible route of the VRD_521_2016 strain in Brazil was through Europe. Despite the uncertainty regarding the country of origin (pp: 0.52), our analysis suggested that viral isolates might have been introduced to Brazil from Spain between the end of 2016 and the beginning of 2017. In addition, we were able to estimate the root of this clade, with the inferred origin in 2013 in Italy (pp: 0.99).

**Figure 6:**
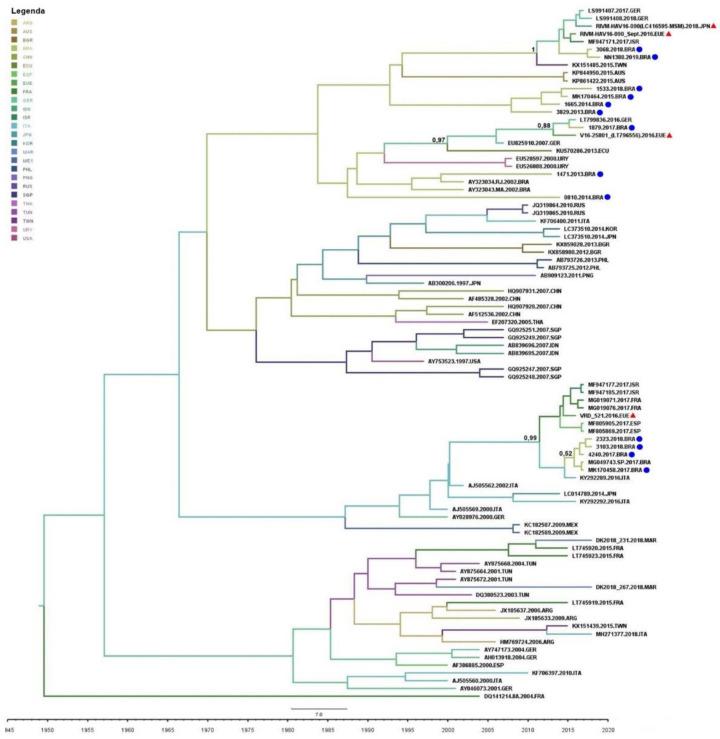
Time-scaled Bayesian MCMC tree of the hepatitis A virus from different countries between the years 1995 and 2020. The European/Asian-related viral strains are marked with a red triangle (▲). The study sequences are marked with a blue dot (●). Branches are colored according to the most probable location of their descendent nodes. The color code is indicated in the upper left legend: ARG = Argentina; AUS = Australia; BGR = Bulgaria; BRA = Brazil; CHN = China; ECU = Ecuador; ESP = Spain; USA = European Union; FRA = France; GER = Germany; IDN = India; ISR = Israel; ITA = Italy; JPN = Japan; KOR = South Korea; MAR = Morocco; MEX = Mexico; PHL = Philippines; PNG = Papua New Guinea; RUS = Russia; SGP = Singapore; THA = Thailand; TUN = Tunisia; TWN = Taiwan; URY = Uruguay; USA = United States of America. Number in the tree represented the pp.

For the Asian and European V16-25801 and RIVM-HAV16-090 strains, our analyses suggested that both were introduced to Brazil through Germany (pp: 1.00 and pp: 0.88, respectively). For the Euro-Asian RIVM-HAV16-090 strain, the probable interval of introduction was the second semester of 2014 and the beginning of 2015, with probable origin in 2011. The strain V16-25801, on the other hand, was possibly introduced to Brazil between the second semester of 2015 and the beginning of 2016, with its probable origin in the year 2000 (pp: 0.97). According to our analysis, it was not possible to estimate the origin of endemic strains circulating in Brazil (clade IV) (Figure 6).

## 4. Discussion

Despite the decline in HAV infections in the last decade in Brazil, an increase in the number of notified HAV cases was observed from 2017 to 2019 among young adults, mainly in São Paulo and Rio de Janeiro cities [17,18], with sexual practices as the most probable route of transmission [6,18].

Our study identified the circulation of three HAV strains that may be associated with the increase in HAV cases in young men in Brazil during the study period. Furthermore, these strains were related to European/Asian outbreaks in MSM.

In this study, Rio de Janeiro’s northern zone held the majority (59.62%; 1.17 cases per 100,000 inhabitants) of HAV cases between 2017 and 2019. This geographical region is the most populous area of the city, with the highest population density (10,189 inhabitants/km²) [26]. Moreover, this region holds the largest number of city slums, areas with high population density with socio-economic, -cultural, and -educational vulnerability, and, in most cases, composed of clusters of small houses that lack potable water and sewage systems [28,29,30]. High-density large agglomerations and low socio-economic conditions facilitate the spread of communicable diseases [1,2,31] and together pose potential risks in the spread of HAV in this region of the city.

It is known that contaminated water and food are the major factors for acquiring HAV infection [1]. However, only a few individuals reported these risk factors (water-borne: 42.31% and food-borne: 44% risks) in this study. In addition, 34.7% of parenteral exposure was observed. Our findings suggest that water-borne, food-borne, and parenteral factors were not the major routes for HAV infection in this study, with interpersonal contact being possibly more relevant. Nevertheless, these factors cannot be excluded and may have played a role as outbreak amplifiers, at least in a portion of the cases.

Other studies have shown that interpersonal contact, including sexual, is a determining factor in some outbreaks [1,3,5,32,33,34]. The predominance of male gender (78.85%) has similarly been reported in other studies [11,13,35,36] and the Brazilian Ministry of Health Viral Hepatitis Bulletin [17,18]. Studies have reported sexual practices as significant risk factors for HAV infection, with the practices of oral, oroanal, and anal sex as important transmission routes [32,33,34,35,37]. Likewise, recent Brazilian studies suggested that the increased HAV incidence in Brazil could be associated with sexual practices between men [10,38]. It is noteworthy that the majority of HAV cases during the outbreak period occurred in sexually active men. This age/gender profile was observed in both the study population and throughout Brazil (Figure 1 and Figure 2). In our analysis, 96% of the infected individuals reported sexual practice, especially oral sex (88.64%) and anal sex (73.91%). A possible explanation is the direct oral–anal contact during sexual activity in an area possibly contaminated with infected feces, leading to contact with high viral titers. Statistical significance was not achieved possibly due to study limitations, such as the lack of complete information in the medical records and/or omission of patient information in the case group. Data obtained here may contribute to elucidating factors related to the recent outbreaks of HAV in adult men in Brazil and throughout the world since the majority (57.69%) of the individuals in this population were MSM (*p* = 0.002). It is important to note that this same profile was observed in outbreaks that occurred in European countries [13].

Despite both biological sexes and sexual orientations reporting the same sexual practices, studies showed that specific sexual practices (such as receptive and insertional oral and anal sex) are more common among MSM than in heterosexuals and women who have sex with women [39,40]. This reinforces that some sexual practices linked to sexual orientation may play a role in acquiring HAV infection in this population. It is also noteworthy that HAV transmission may be more related to the practice itself than to the sexual orientation of the individual since non-MSM individuals have also been infected.

The relative risk analysis showed that individuals in our study who traveled to endemic regions had a 7.19-fold (*p* < 0.01) increased risk of becoming infected with HAV. A review published in 2018 by Jacobsen reported that travel to endemic regions is related to high rates of HAV infection [2]. A similar finding was observed by Chuffi and colleagues in a recent study conducted in São Paulo, where having traveled in the last 2 months before symptoms was related to HAV positivity [38]. It is important to highlight here that several MSM patients in our study reported trips to São Paulo, which was the epicenter for the HAV outbreak among Brazilian men [10,17,18].

Moreover, it is worth mentioning that 25% of individuals also had at least one STI (HIV and/or syphilis). All were males and MSM. This high prevalence of STIs may be related to the expressive rates of unprotected sex in the study population (78.26%), further reinforcing sexual practices and their link to the transmission of HAV and other STIs. Several studies also reported the association of HAV co-infection with HIV and syphilis through unprotected sexual practices [11,32,33,34,36].

Phylogenetic analysis revealed that all samples belonged to the subgenotype IA, the most prevalent genotype circulating in Brazil, as stated by previous studies [20,41]. However, the majority (30/35; 85.7%) of the 2017–2019 circulating strains were related to MSM outbreaks in Europe/Asia, most of which clustered with the VRD_521_2016 strain (23/35; 65.8%), followed by RIMV-HAV16-090 (6/35; 17.1%), and less frequently, V16-25801 (1/35; 2.8%). A similar proportion has been observed in a study conducted in Sâo Paulo [38] and in European HAV outbreaks, where the VRD_521_2016 strain was present in most cases [13]. Moreover, the strain VRD_521_2016 was identified in sewage samples from São Paulo during the outbreak [42]. It demonstrated that, although this strain may have been spread through sexual practices among MSM, it is not restricted to this route or this group. Once in the environment, it may easily reach new hosts through contaminated water and food. Our findings showed that the imported viral strains may have been responsible for the increase in the number of HAV cases in Rio de Janeiro city and other cities in the southeastern and southern regions of Brazil. Other studies have acknowledged the introduction of new viral variants as possible sources for new outbreaks, especially associated with less common transmission routes [1,43,44].

In a previous study performed by our group, based on the first outbreak of HAV strains sequenced in Brazil, the introduction of VRD_521_2016 to this country occurred most likely during the Olympics and Paralympics games [15]. The present study reinforces this previously published finding with more robust data, as a larger number of sequences were analyzed along with the inclusion of samples from before, during, and after the abrupt increase in HAV infection in the city of Rio de Janeiro. In addition, our analyses were able to identify other viral strains related to international MSM outbreaks in Brazil, and through Bayesian analyses, inferred the most recent common ancestor and their dispersal routes, with mean evolutionary rate, estimated in 1.05 × 10^−5^ substitutions/site/year. This evolutionary rate is consistent with other studies on HAV genotype IA [15,45,46].

Regarding the outbreak strains found in this study, VRD_521_2016 was first identified in 2016 in the United Kingdom [34,46,47]. Our analysis dated its probable origin in 2013 in Italy, imported to Brazil through Spain, and introduced here in the same period as the Olympic/Paralympic Games (2016). The second outbreak strain, V16-25801, was first notified in Italy in 2014 [48], and the third strain, RIVM-HAV16-090, in Asia in 2015 and the Netherlands in 2016 [13,14,46]. Our analyses showed that their probable origin was the European continent, in 2000 and 2011, respectively. Both might have been introduced in Brazil through Germany in the same period as the World Cup (2014) and the Olympic/Paralympic Games (2016). According to the World Health Organization, sports events involving a large flow of people from different continents with diverse immunological backgrounds are often associated with an increased risk of the spread of communicable diseases and the introduction of imported pathogens [31]. These events hosted in Rio de Janeiro, with an expressive increase in the number of tourists, possibly led to the introduction of these HAV strains in Brazil and were responsible for the increase in the number of cases among young adults between 2017–2019.

This study has some limitations: despite the Brazilian epidemiological context suggesting that sexual practices may have played a role in the increase in the number of HAV cases between 2017–2019, no statistical support was obtained in this study to validate this hypothesis. As mentioned, this may be explained by the lack of complete information in the medical records. In agreement, some variables, such as the number of sexual partners and raw vegetable consumption, were possibly biased due to inadequate data filling (recall bias and/or deliberate omission of information by the respondents). It is possible that questions involving sexual aspects may have been omitted, thus limiting statistical analyses. Another point was the scarcity of Brazilian HAV sequences published in the databases from the last 10 years, partially limiting spatio-temporal analyses. Despite these limitations, this study provided relevant epidemiological and molecular data on HAV infection in Brazil, highlighting the importance of monitoring this infection in adults and key populations.

## 5. Conclusions

In conclusion, the northern zone of Rio de Janeiro held the majority of HAV cases in the city, most of them among young adult MSM, with sexual practices as a possible transmission factor. Traveling to endemic areas as well as the intense tourism in Brazil due to sports events during HAV European MSM outbreaks possibly played a role in introducing and disseminating HAV outbreak strains. Our study highlights the need for health policies to improve access to HAV vaccination for adult groups and key populations and reinforces the importance of monitoring the introduction of new pathogens in Brazil. In addition, the implementation of educational measures targeted to key populations may be useful to prevent the dissemination of HAV and other STIs.

## Figures and Tables

**Table 1:** Descriptive sociodemographic characteristics (2017–2019).

Variables	Total (*n* = 139)		Hepatitis A (Case Group) (*n* = 52)		Control Group (*n* = 87)		*p*-Value *
	*n*	(*%*)	*n*	(*%*)	*n*	(*%*)	
**Biological Sex**							**0.024**
Feminine	47	(33.81)	11	(21.15)	36	(41.38)	
Masculine	92	(66.19)	41	(78.85)	51	(58.62)	
**Sexual Orientation**							**0.002**
Heterosexual Women	41	(29.5)	8	(15.38)	33	(37.93)	
WSW ^1^	6	(4.32)	3	(5.77)	3	(3.45)	
Heterosexual Men	38	(27.34)	11	(21.15)	27	(31.03)	
MSM ^2^	54	(38.85)	30	(57.69)	24	(27.59)	
**Age Groups**							**<0.001**
20 to 29 years	32	(23.02)	24	(46.15)	8	(9.2)	
30 to 39 years	42	(30.22)	20	(38.46)	22	(25.29)	
40 to 59 years	36	(25.9)	7	(13.46)	29	(33.33)	
60 years or more	29	(20.86)	1	(1.93)	28	(32.18)	
**Mean age**	40.03 ± 12.97 (20 to 77 years)	-	32.19 ± 10.54 (20 to 73 years)	-	44.71 ± 12.03 (22 to 77 years)	-	**<0.001**
**Planning Area ^3^**							**0.001**
North ^†^	2.23	(42.45)	1.17	(59.62)	1.06	(32.18)	
South ^†^	0.69	(6.47)	0.08	(1.92)	0.6 2	(9.2)	
West ^†^	1.72	(28.78)	0.34	(15.38)	1.38	(36.78)	
Central ^†^	0.85	(7.91)	0.54	(13.46)	0.31	(4.6)	
Neighboring cities **^a^**	0.75	(14.39)	0.18	(9.62)	0.56	(17.24)	

Note: *n* = participant’s number; * Chi-square test (*p* < 0.05); ^1^ men have sex with men; ^2^ women have sex with women; ^3^ Cases per 100,000 inhabitants; † total population per area = 2,646,515, 1,297,721, 2,321,863, and 1,297,721, respectively [26]; ^a^ Duque de Caxias, Nova Iguaçu, and São Gonçalo (total population = 2,651,033) [26].

**Table 2:** Patients’ socio-behavioral characteristics: HAV exposure factors.

HAV Exposure Categories	Total (*n* = 139) ^†^		Hepatitis A (Case Group) (*n* = 52) ^†^		Control Group (*n* = 87) ^†^		*p*-Value *	Unadjusted		Adjusted	
	* n *	(*%*)	* n *	(*%*)	* n *	(*%*)		OR (95% CI)	*p*-Value **	OR (95% CI)	*p*-Value **
**Parenteral factor**							**0.056**				
**Yes**	**63**	**(45.99)**	**18**	**(34.62)**	**45**	**(52.94)**		**0.42** **(0.17;1.02)**	**0.1254**	**0.46** **(0.18; 1.17)**	**0.1076**
**No**	**74**	**(54.01)**	**34**	**(65.38)**	**40**	**(47.06)**		**1.00**		**1.00**	
Injectable Remedies							0.818				
Yes	21	(15.44)	9	(17.31)	12	(14.29)		-	-	-	-
No	115	(84.56)	43	(82.69)	72	(85.71)		-	-	-	-
Inhalable Drugs							1.000				
Yes	14	(10.29)	5	(9.62)	9	(10.71)		-	-	-	-
No	122	(89.71)	47	(90.38)	75	(89.29)		-	-	-	-
Injectable Drugs							0.698				
Yes	2	(1.47)	0	(0)	2	(2.38)		-	-	-	-
No	134	(98.53)	52	(100)	82	(97.62)		-	-	-	-
Tattoos							0.751				
Yes	13	(9.56)	6	(11.54)	7	(8.33)		-	-	-	-
No	123	(90.44)	46	(88.46)	77	(91.67)		-	-	-	-
Piercings							0.662				
Yes	10	(7.41)	5	(9.62)	5	(6.02)		-	-	-	-
No	125	(92.59)	47	(90.38)	78	(93.98)		-	-	-	-
Dental treatment							1.000				
Yes	24	(17.65)	9	(17.31)	15	(17.86)		-	-	-	-
No	112	(82.35)	43	(82.69)	69	(82.14)		-	-	-	-
Blood transfusion							0.063				
Yes	8	(5.93)	0	0	8	(9.41)		-	-	-	-
No	127	(94.07)	50	(100)	77	(90.59)		-	-	-	-
**Food-borne factors**							**0.101**				
**Yes**	**38**	**(34.86)**	**22**	**(44)**	**16**	**(27.12)**		**1.52** **(0.62–3.82)**	**0.2891**	**1.71** **(0.62–4.82)**	**0.2992**
**No**	**71**	**(65.14)**	**28**	**(56)**	**43**	**(72.88)**		**1.00**		**1.00**	
Raw meat consumption							0.632				
Yes	29	(27.36)	15	(30.61)	14	(24.56)		-	-	-	-
No	77	(72.64)	34	(69.39)	43	(75.44)		-	-	-	-
Raw vegetable consumption							0.018 *				
Yes	9	(9)	8	(17.39)	1	(1.85)		-	-	-	-
No	91	(91)	38	(82.61)	53	(98.15)		-	-	-	-
Well water and/or cistern water consumption							0.405				
Yes	6	(7.23)	4	(11.43)	2	(4.17)		-	-	-	-
No	77	(92.77)	31	(88.57)	46	(95.83)		-	-	-	-
**Water-borne factors**							**0.410**				
**Yes**	**40**	**(37.38)**	**22**	**(42.31)**	**18**	**(32.73)**		**1.53** **(0.64–3.70)**	**0.9041**	**0.97** **(0.34–2.62)**	**0.9464**
**No**	**67**	**(62.62)**	**30**	**(57.69)**	**37**	**(67.27)**		**1.00**		**1.00**	
Floodwater exposure							0.786				
Yes	12	(11.88)	7	(13.73)	5	(10)		-	-	-	-
No	89	(88.12)	44	(86.27)	45	(90)		-	-	-	-
Beach bathing							1.000				
Yes	19	(18.27)	10	(19.23)	9	(17.31)		-	-	-	-
No	85	(81.73)	42	(80.77)	43	(82.69)		-	-	-	-
River and/or lake bathing							1.000				
Yes	8	(7.77)	4	(7.69)	4	(7.84)		-	-	-	-
No	95	(92.23)	48	(92.31)	47	(92.16)		-	-	-	-
Well water supply							0.263				
Yes	3	(3.03)	3	(5.88)	0	(0)		-	-	-	-
No	96	(96.97)	48	(94.12)	48	(100)		-	-	-	-
Cistern water supply							0.493				
Yes	2	(2.08)	2	(4.08)	0	(0)		-	-	-	-
No	94	(97.92)	47	(95.92)	47	(100)		-	-	-	-
Piped water supply							1.000				
Yes	89	(92.71)	46	(92)	43	(93.48)		-	-	-	-
No	7	(7.29)	4	(8)	3	(6.52)		-	-	-	-
Sewage system							0.752				
Yes	90	(93.75)	46	(92)	44	(95.65)		-	-	-	-
No	6	(6.25)	4	(8)	2	(4.35)		-	-	-	-
**Sexual Factors**							**0.062**				
**Yes**	**114**	**(88.37)**	**48**	**(96)**	**66**	**(83.54)**		**1.81** **(0.33–13.59)**	**0.9178**	**0.88** **(0.15–6.99)**	**0.8902**
**No**	**15**	**(11.63)**	**2**	**(4)**	**13**	**(16.46)**		**1.00**		**1.00**	
Oral Sex							0.167				
Yes	72	(81.82)	39	(88.64)	33	(75)		-	-	-	-
No	16	(18.18)	5	(11.36)	11	(25)		-	-	-	-
Oroanal sex							0.826				
Yes	32	(46.38)	19	(44.19)	13	(50)		-	-	-	-
No	37	(53.62)	24	(55.81)	13	(50)		-	-	-	-
Anal Sex							0.323				
Yes	58	(68.24)	34	(73.91)	24	(61.54)		-	-	-	-
No	27	(31.76)	12	(26.09)	15	(38.46)		-	-	-	-
≥3 sexual partners							0.004 *				
Yes	38	(42.22)	11	(25.58)	27	(57.45)		-	-	-	-
No	52	(57.78)	32	(74.42)	20	(42.55)		-	-	-	-
Vaginal Sex							0.082				
Yes	60	(82.19)	16	(100)	44	(77.19)		-	-	-	-
No	13	(17.81)	0	(0)	13	(22.81)		-	-	-	-
Unprotected sex							0.980				
Yes	89	(79.46)	36	(78.26)	53	(80.3)		-	-	-	-
No	23	(20.54)	10	(21.74)	13	(19.7)		-	-	-	-
**Travel to endemic region**							0.001 *				
**Yes**	**19**	**(18.81)**	**15**	**(34.09)**	**4**	**(7.02)**		**6.90** **(2.01–32.16)**	<0.01 **	**7.19** **(1.93–36.04)**	<0.01 **
**No**	**82**	**(81.19)**	**29**	**(65.91)**	**52**	**(92.98)**		**1.00**		**1.00**	

Note: *n* = participants’ number; OR = Odds ratio; * Chi-square test (*p* < 0.05); ** Statistically significant in OR (*p* < 0.05); ^†^ The values can be less than the total value due to lack of information.

## Data Availability

Data kindly provided by the Brazilian Ministry of Health can also be found on the government page: DataSUS-TabNet, Notifiable Diseases Information System, http://www2.datasus.gov.br/DATASUS/index.php?area=0203&id=29878153, accessed on 17 December 2021.

## Supplementary Materials

The following supporting information can be downloaded at: https://www.mdpi.com/article/10.3390/v14020303/s1, Table S1: GenBank access numbers of references strains. Table S2: GenBank access numbers from study strains.
