# Supplementary material for: Increase in Hepatitis A Cases Linked to Imported Strains to Rio de Janeiro, Brazil: A Cross-Sectional Study
Source: Viruses. 2022 Feb 1;14(2):303. doi: 10.3390/v14020303 (PMC8874517; doi:10.3390/v14020303)
Supplement: Supplementary file 1 [file viruses-14-00303-s001.zip › Supplementary table 1.pdf]

Supplementary Table S1. Genbank access numbers of references strains.

Access number	Country	Years	Subgenotypes
AY028976	Germany	2000	IA
AY046073	Germany	2001	IA
AH013918	Germany	2004	IA
EU825910	Germany	2007	IA
LT799836	Germany	2016	IA
LS991407	Germany	2017	IA
LS991408	Germany	2018	IA
HM769724	Argentina	2006	IA
JX185637	Argentina	2006	IA
JX185633	Argentina	2009	IA
AY322956	Brazil	2002	IA
AY322961	Brazil	2002	IA
AY322981	Brazil	2002	IA
AY323018	Brazil	2002	IA
AY323034	Brazil	2002	IA
AY323045	Brazil	2002	IA
MG049743	Brazil	2017	IA
KX859028	Bulgaria	2013	IA
AF485328	China	2002	IA
AF512536	China	2002	IA
HQ907928	China	2007	IA
LC373510	South Korea	2014	IA
KU570286	Ecuador	2013	IA
AF386885	Spian	2000	IA
MF805869	Spian	2017	IA
MF805905	Spian	2017	IA
LT796556	Europe	2016	IA
AB793725	Philippines	2012	IA
LT745919	France	2015	IA
LT745920	France	2015	IA
MG019071	France	2017	IA
MG019076	France	2017	IA
AB839669	India	2007	IA
AB839695	India	2007	IA
MF947171	Israel	2017	IA
MF947177	Israel	2017	IA
MF947185	Israel	2017	IA
AJ505560	Italy	2000	IA
AJ505569	Italy	2000	IA

AJ505562	Italy	2002	IA
KF706400	Italy	2011	IA
KY292289	Italy	2016	IA
KY292292	Italy	2016	IA
MH271377	Italy	2018	IA
AB300206	Japan	1997	IA
LC014789	Japan	2014	IA
LC373510	Japan	2014	IA
LC416595	Japan	2018	IA
DK2018231	Morocco	2018	IA
DK2018267	Morocco	2018	IA
KC182588	Mexico	2009	IA
AB909123	Papua New Guinea	2011	IA
JQ319865	Russia	2010	IA
GQ925247	Singapore	2007	IA
GQ925249	Singapore	2007	IA
EF207320	Thailand	2005	IA
KX151439	Taiwan	2015	IA
KX151485	Taiwan	2915	IA
DQ380523	Tunisia	2003	IA
AY875668	Tunisia	2004	IA
AY875668	Tunisia	2004	IA
EU526088	Uruguay	2008	IA
DQ141214	France	2004	IIA
