# Supplementary material for: Increase in Hepatitis A Cases Linked to Imported Strains to Rio de Janeiro, Brazil: A Cross-Sectional Study
Source: Viruses. 2022 Feb 1;14(2):303. doi: 10.3390/v14020303 (PMC8874517; doi:10.3390/v14020303)
Supplement: Supplementary file 1 [file viruses-14-00303-s001.zip › Supplementary table 2.pdf]

**Supplementary Table S2.** Genbank access numbers from study strains.

| Sample Name | Access number | Country | Year | Genotype |
|---|---|---|---|---|
| 0221.2013.BRA | MW404263 | Brazil | 2013 | IA |
| 0718.2013.BRA | MW404268 | Brazil | 2013 | IA |
| 1471.2013.BRA | MW404283 | Brazil | 2013 | IA |
| 2150.2013.BRA | MW404290 | Brazil | 2013 | IA |
| 3829.2013.BRA | MW404299 | Brazil | 2013 | IA |
| 0810.2014.BRA | MW404270 | Brazil | 2014 | IA |
| 1665.2014.BRA | MW404286 | Brazil | 2014 | IA |
| 1853.2014.BRA | MW404288 | Brazil | 2014 | IA |
| 3225.2014.BRA | MW404297 | Brazil | 2014 | IA |
| 3253.2014.BRA | MW404298 | Brazil | 2014 | IA |
| MK170460.2015.BRA[¶] | MK170460[¶] | Brazil | 2015 | IA |
| MK170461.2015.BRA[¶] | MK170461[¶] | Brazil | 2015 | IA |
| MK170462.2015.BRA[¶] | MK170462[¶] | Brazil | 2015 | IA |
| MK170463.2015.BRA[¶] | MK170463[¶] | Brazil | 2015 | IA |
| MK170464.2015.BRA[¶] | MK170464[¶] | Brazil | 2015 | IA |
| MK170465.2015.BRA[¶] | MK170465[¶] | Brazil | 2015 | IA |
| MK170466.2015.BRA[¶] | MK170466[¶] | Brazil | 2015 | IA |
| MK170458.2017.BRA[¶] | MK170458[¶] | Brazil | 2017 | IA |
| MK170459.2017.BRA[¶] | MK170459[¶] | Brazil | 2017 | IA |
| 1879.2017.BRA | MW404289 | Brazil | 2017 | IA |
| 4240.2017.BRA | MW404300 | Brazil | 2017 | IA |
| 0076.2018.BRA | MW404261 | Brazil | 2018 | IA |
| 0199.2018.BRA | MW404303 | Brazil | 2018 | IA |
| 0278.2018.BRA | MW404264 | Brazil | 2018 | IA |
| 0337.2018.BRA | MW404265 | Brazil | 2018 | IA |
| 0411.2018.BRA | MW404266 | Brazil | 2018 | IA |
| 0498.2018.BRA | MW404267 | Brazil | 2018 | IA |
| 0758.2018.BRA | MW404269 | Brazil | 2018 | IA |
| 0927.2018.BRA | MW404272 | Brazil | 2018 | IA |
| 0943.2018.BRA | MW404273 | Brazil | 2018 | IA |
| 0945.2018.BRA | MW404274 | Brazil | 2018 | IA |
| 0962.2018.BRA | MW404275 | Brazil | 2018 | IA |
| 1061.2018.BRA | MW404276 | Brazil | 2018 | IA |
| 1110.2018.BRA | MW404277 | Brazil | 2018 | IA |
| 1191.2018.BRA | MW404278 | Brazil | 2018 | IA |
| 1294.2018.BRA | MW404279 | Brazil | 2018 | IA |
| 1494.2018.BRA | MW404284 | Brazil | 2018 | IA |
| 1533.2018.BRA | MW404285 | Brazil | 2018 | IA |
| 1794.2018.BRA | MW404287 | Brazil | 2018 | IA |
| 2152.2018.BRA | MW404291 | Brazil | 2018 | IA |
| 2323.2018.BRA | MW404292 | Brazil | 2018 | IA |
| 2472.2018.BRA | MW404293 | Brazil | 2018 | IA |

| | | | | |
|---|---|---|---|---|
| 3068.2018.BRA | MW404294 | Brazil | 2018 | IA |
| 3103.2018.BRA | MW404295 | Brazil | 2018 | IA |
| 3158.2018.BRA | MW404296 | Brazil | 2018 | IA |
| NN4810.2018.BRA | MW404301 | Brazil | 2018 | IA |
| NN4951.2018.BRA | MW404302 | Brazil | 2018 | IA |
| 0196.2019.BRA | MW404262 | Brazil | 2019 | IA |
| 0278.2019.BRA | MW404271 | Brazil | 2019 | IA |
| 1304.2019.BRA | MW404280 | Brazil | 2019 | IA |
| NN1388.2019.BRA | MW404281 | Brazil | 2019 | IA |
| NN1391.2019.BRA | MW404282 | Brazil | 2019 | IA |

¶Sequences previous published by Mello et al, 2019[15]
